# Design and characteristics of the national Danish injury cohort (NDIC)

**DOI:** 10.1007/s10654-026-01413-2

**Published:** 2026-06-08

**Authors:** Mette Rasmussen, Sofie Kruckow, Isabelle Pascale Mairey, Anne Illemann Christensen

**Affiliations:** National Institute of Public Health, University of Southern Denmark, Copenhagen, Denmark

**Keywords:** Injury, Accident, Register-based, Cohort, Unnatural death, Denmark

## Abstract

The National Danish Injury Cohort (NDIC) is a nationwide, register-based cohort established to examine the incidence of total and specific injuries in Denmark and to provide insights into causes and consequences. Comprehensive individual-level information for this cohort is stored in the NDIC dataset, serving as a foundation for analyses of injury determinants and supporting the planning and evaluation of preventive measures aimed at reducing injury-related harm. Individuals registered in the Danish National Patient Register with an injury from 2010 onwards are included in the dataset. Additionally, it comprises persons in the Cause of Death Register whose deaths was attributed to accidents, violence, or suicide. The cohort is currently updated through 2022 and contains information on more than 7.2 million primary injury contacts and 26 thousand accident-related deaths. Analyses based on NDIC demonstrated substantial demographic and geographical disparities in the incidence rates of injuries. Overall, men exhibited higher rates of both injury incidents and injury‑related mortality compared with women. Marked differences were observed when stratifying by sex and age, as well as by region of residence. NDIC offers a solid data foundation facilitating research into injury trends, causes, and impacts, including disparities across social groups and geographic areas. It presents unique opportunities to explore novel research ideas to boost injury prevention, improve targeting of interventions, and reduce health inequalities. Strengthening this research area will help further reinforce NDIC’s role in injury surveillance and evidence‑based policymaking.

**Supplementary Information:**

The online version contains supplementary material available at https://doi.org/10.1007/s10654-026-01413-2.

## Introduction

Injuries remain a major public health issue in Denmark, as in many other countries [1, 2], and can have profound short- and long-term consequences for the affected individuals and their relatives. Moreover, they result in considerable morbidity and mortality and impose substantial societal and economic burdens [1]. In this paper, the term “injury incident” is used broadly to include unintentional injury events as well as intentional injury events such as violence, suicide, suicide attempts, and self-harm. The term “injury” refers to any physical harm resulting from these events. Injury related mortality includes death from accident, violence, and suicide.

In Denmark, injury incidents are a major cause of hospital admissions and premature death, particularly among adolescents and young adults [3, 4], and generate substantial demand for prehospital emergency services and consultations in primary care. The burden of injury incidents is considerable. Beyond affecting injured individuals and their families, injury incidents can lead to substantial losses of healthy and productive life years. This places significant pressure on the healthcare system and generates major societal costs through medical treatment and reduced workforce productivity [5, 6]. Understanding the mechanisms behind these events is not only a scientific challenge but also a societal imperative, as preventing such incidents is crucial for individual well-being and for reducing the burden on society across sectors.

Research into prevalence and distribution of injuries provides the foundation for effective prevention strategies, policy development, and resource prioritization [7]. In a Danish context, this work is supported by a long tradition of systematic data collection and analysis. The former Danish Injury Register originally based on data from five emergency departments between 1998 and 2009 [8, 9], has played a central role in this effort. However, following the discontinuation of the register in 2009, the documentation of injury incidents has received considerably less attention on a national level.

Injury research also carries broader implications for social equity. Previous research has demonstrated substantial sociodemographic disparities, with marked differences in incidence rates across age groups, income levels, educational attainment, and geographical regions [5, 10–12]. Identifying and communicating such disparities can help inform targeted interventions and policies that promote safety for all segments of the population.

Moreover, Denmark contributes to international efforts to reduce injury-related harm. Through participation in the European Injury Data Base (IDB-EU) coordinated by EuroSafe and supported by the EU, Danish researchers share insights and data with counterparts across the EU [13]. This collaboration enhances the collective understanding of injury patterns and supports the development of cross-national strategies for prevention of injury incidents.

Injury research relies on systematic data collection, transparent case definitions, appropriate study designs, and rigorous statistical analyses to minimise bias and confounding. Robust methodological frameworks enable researchers to move beyond anecdotal observations and generate reproducible insights that can inform public health interventions, and other safety regulations [7].

The National Danish Injury Cohort (NDIC) was established to facilitate dissemination of data and knowledge on the occurrence, causes, and consequences of injury incidents to a wide range of stakeholders, including IDB-EU. This type of research is necessary to maintain surveillance and analytical capabilities.

### Objective

This paper aims to describe the methodological framework of NDIC, to outline the underlying data structure and content, and to present the methodological considerations underpinning the registry-based identification of hospital and death records related to injury incidents. In addition, initial results are presented showing trends in injury incidence rates across predefined strata of the population from 2010 onwards. Together, these elements highlight the scope and development of the NDIC and illustrate its potential for future analytical applications.

## Cohort description

Denmark has a long-standing tradition of comprehensive data collection through nationwide social, administrative, and health registries, made possible by using the unique Civil Personal Register numbers assigned to all residents with a permanent address in Denmark. The structure, content, and applications of these registries—particularly in epidemiological research—have been described in detail elsewhere [14–16]. In short, all residents in Denmark are assigned a Civil Personal Register number at birth or upon immigration, and the number is used as the key identifier in all interactions in administrative records including the healthcare system, enabling accurate linkage of data across central registries [15]. Individuals without permanent residence—such as foreign workers and temporary visitors—are registered using either a non‑resident Civil Personal Register numbers or, in the case of tourists who require healthcare, a temporary replacement identifier. Hence, injury incidents involving these individuals are also captured within the cohort. Through tax funding, Danish citizens have access to healthcare that is free at the point of use, reducing socioeconomic barriers to seeking care and thereby supporting the completeness of registry-based injury data [15].

### The national Danish injury dataset

While several data sources provide valuable insights into specific types of injury incidents, they are often limited in geographic range, scope or focus, and there is currently no unified entry point or comprehensive overview. Statistics Denmark has published a guide indicating where injury-related data can be found [17]. However, this list is not exhaustive and does not offer a complete overview of available sources.

NDIC offers a uniquely extensive and integrated framework for Danish injury surveillance and research. Its primary purpose is to establish a robust foundation for the systematic monitoring of injury incidents by utilising existing national registers. NDIC draws on data from the Danish National Patient Register (DNPR) [18] from 2010 onwards, capturing all hospital contacts, including emergency department visits, thereby ensuring nationwide coverage of injuries recorded within routine administrative health systems. Injury‑related deaths are identified through the Danish Register of Causes of Death [19]. By linking these data on the individual level with other central registers, NDIC enables consistent identification of injury incidents, standardised classification across time and regions, and comprehensive assessment of injury incidents patterns, determinants, and outcomes, including long‑term consequences of severe injuries. This integrated design supports both broad epidemiological assessments and more focused thematic studies across diverse population groups.

### Organisation and data security

NDIC is organisationally located within the National Institute of Public Health, University of Southern Denmark. Data is stored in the NDIC dataset and processed exclusively on a secure server maintained by Statistics Denmark. Access to Danish central registers is granted by Statistics Denmark and The Danish Health Data Authorities. Data are provided for research purposes in a pseudonymous and secure form.

### Study population

NDIC includes individuals in Denmark who, from 2010 onwards, experienced an injury incident that either resulted in death or required hospital treatment, including visits to emergency departments. While hospital contacts also include individuals without permanent residence in Denmark, deaths can only be identified for persons who are registered as residents in the country.

### Data sources

NDIC relies exclusively on routinely collected data from DNPR [18], death certificates [19], and other administrative registers such as population statistics [20], education registers [21], and labour market and income registers [22]. No new data collecting is undertaken.

### The Danish register of causes of death

In the Danish Register of Causes of Death [19], each death is categorised by the manner of death as either natural, accident, suicide, violence/homicide, or undetermined if the circumstances are unclear after an autopsy has been performed. The underlying cause of death refers to the medical condition or injury that initiated the chain of event leading to death (e.g. diabetes or fall) and is mandatory to register. The immediate cause of death is the cause directly leading to death (e.g. cardiac arrest or respiratory failure). Data are collected from death certificates completed by physicians during post-mortem examinations and reported to the Danish Health Data Authority through an electronic reporting system. For unnatural deaths, that is if the manner of death is accident, suicide or violence/homicide, the information is validated by the Danish Health Data Authority.

### The Danish national patient register (DNPR)

Hospital contacts related to injury incidents were obtained from DNPR [23], which includes records from inpatient and outpatient visits across public and private hospitals, encompassing somatic as well as psychiatric care records [18]. The data contains information on the circumstances of the injury incident and the injuries sustained, including contextual and event-related aspects, as well as diagnostic codes describing the nature and severity of the injuries. An action diagnosis is assigned, referring to the condition that prompted the patient’s contact with the healthcare system. It reflects the most acute or treatment-requiring condition during hospital admissions, outpatient visits, and emergency department encounters, making it central to the clinical care pathway.

### Coding systems

In Denmark, injury data is coded using the Danish Medical Classification System (SKS) [24, 25], which incorporate internationally recognized systems such as the International Classification of Diseases (ICD) [25] and NOMESCO’s Classification of External Causes of Injuries (NCECI) [7], ensuring consistency and comparability across regions and over time.

### International classification system for diseases and causes of death

The ICD classification [25], based on the WHO’s international classification system for diseases and causes of death [26], is used to record diagnoses and health-related conditions in DNPR. Throughout the study period (2010 onwards) the tenth version (ICD-10) has been used.

For accident-related coding, specific ICD-10 codes are applied to classify injuries, as a result of accidents, violence, and other external causes. The codes, found in Chapter XIX (DS00–DT98) cover a wide range of injuries and conditions caused by external factors. These include injuries to various body parts (e.g., head, neck, chest, abdomen, back, arms, and legs), as well as burns, corrosions, frostbite, and poisonings from both medical and non-medical substances. The codes also encompass complications following surgical and medical procedures, and long-term consequences of injuries and poisonings. Codes from Chapter XX (DX60–Y09), are used to classify and describe intentional self-harm, assaults, and other external causes of morbidity and mortality. These codes include cases of self-injury, suicide attempts, and various forms of violence events, including physical, sexual, and psychological abuse.

The purpose of these codes is to provide detailed information about the circumstances of the accident, which is essential for both clinical care and epidemiological research.

### NOMSECO classification of external causes of injuries

To establish a common health terminology and ensure consistency and comparability of health data across countries, the Nordic Medico-Statistical Committee (NOMESCO) developed the NCECI codes. This standardised classification system is used across Nordic countries to record and analyse external causes of injury incidents and injuries [7].

The NCECI coding system provides a hierarchical structure for classifying various types of injury incidents and injuries, in which each code represents a specific type of incident. The system enables detailed recording of injury-related information, including codes on activity (the victim’s activity at the time of the event, e.g. sports, paid work), place of occurrence (where the event happened, e.g. home, school, road), and mode of injury (how the injury occurred, e.g. fall, transportation accident).

In Denmark, NCECI codes (version 4) [7] have been translated and integrated into SKS, represented by codes beginning with “EU” (EUA–EUZ).

### Identification of injury-related contacts

**Fig. 1:**
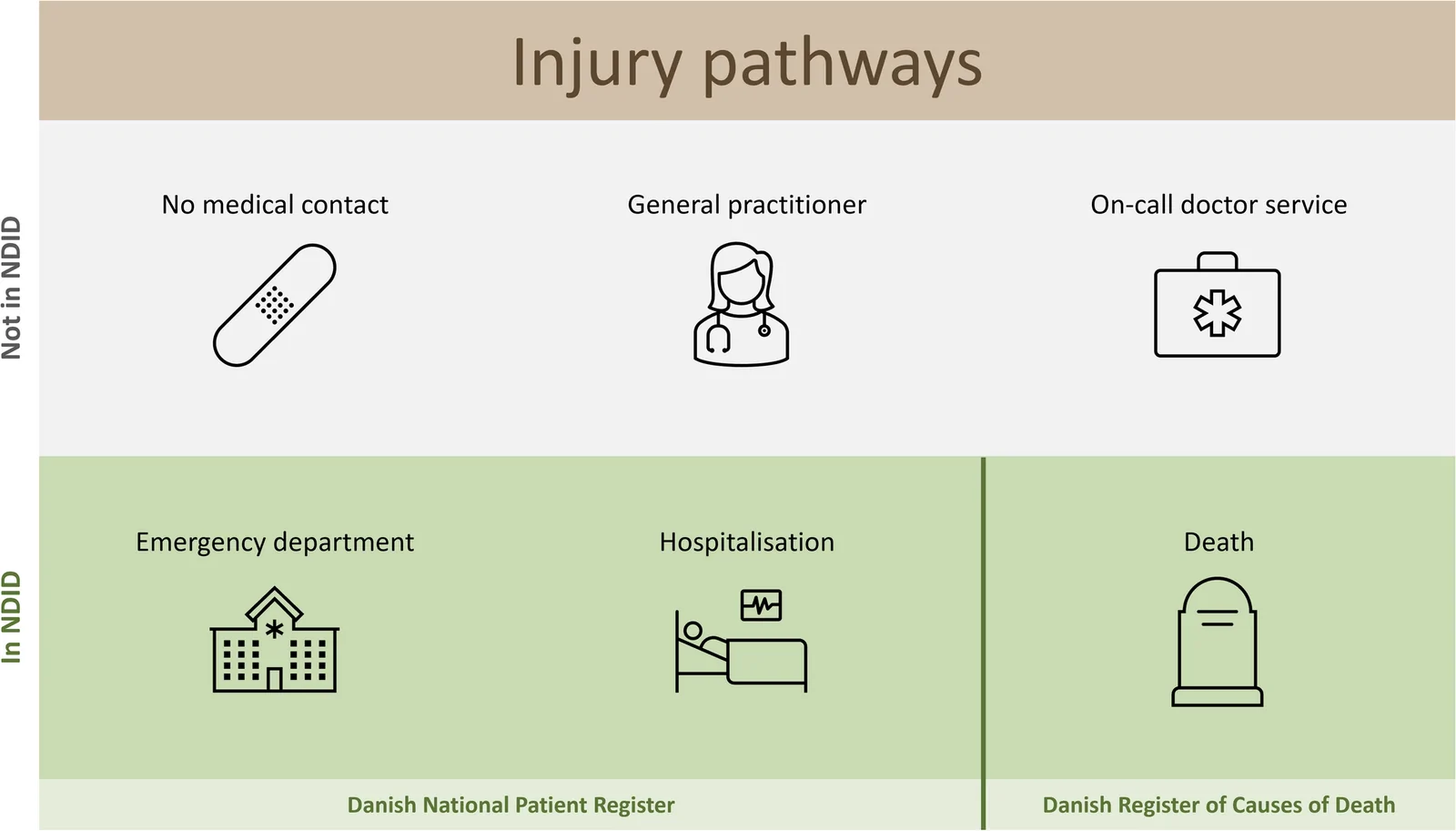
Possible pathways through the healthcare system following an injury incident The figure illustrates six potential pathways following an injury: (1) no medical contact, (2) consultation with a general practitioner, (3) contact with a public or private on-call doctor service (including ambulance dispatch resulting in on-site assessment and treatment without hospital transport), (4) emergency department visit (including hospital-based urgent care clinics), (5) hospital admission or outpatient treatment, and (6) death. NDIC includes outcomes 4–6, which are captured through the DNPR and the Danish Register of Causes of Death. Outcomes 1–3 are not included in the cohort.

Individuals exposed to injury incidents may navigate the healthcare system in various ways. In a Danish context, we have identified six distinct pathways that exemplify how such trajectories may unfold. These pathways are not mutually exclusive, and individuals may transition between them or follow elements of more than one, see Fig. 1.

**Fig. 2:**
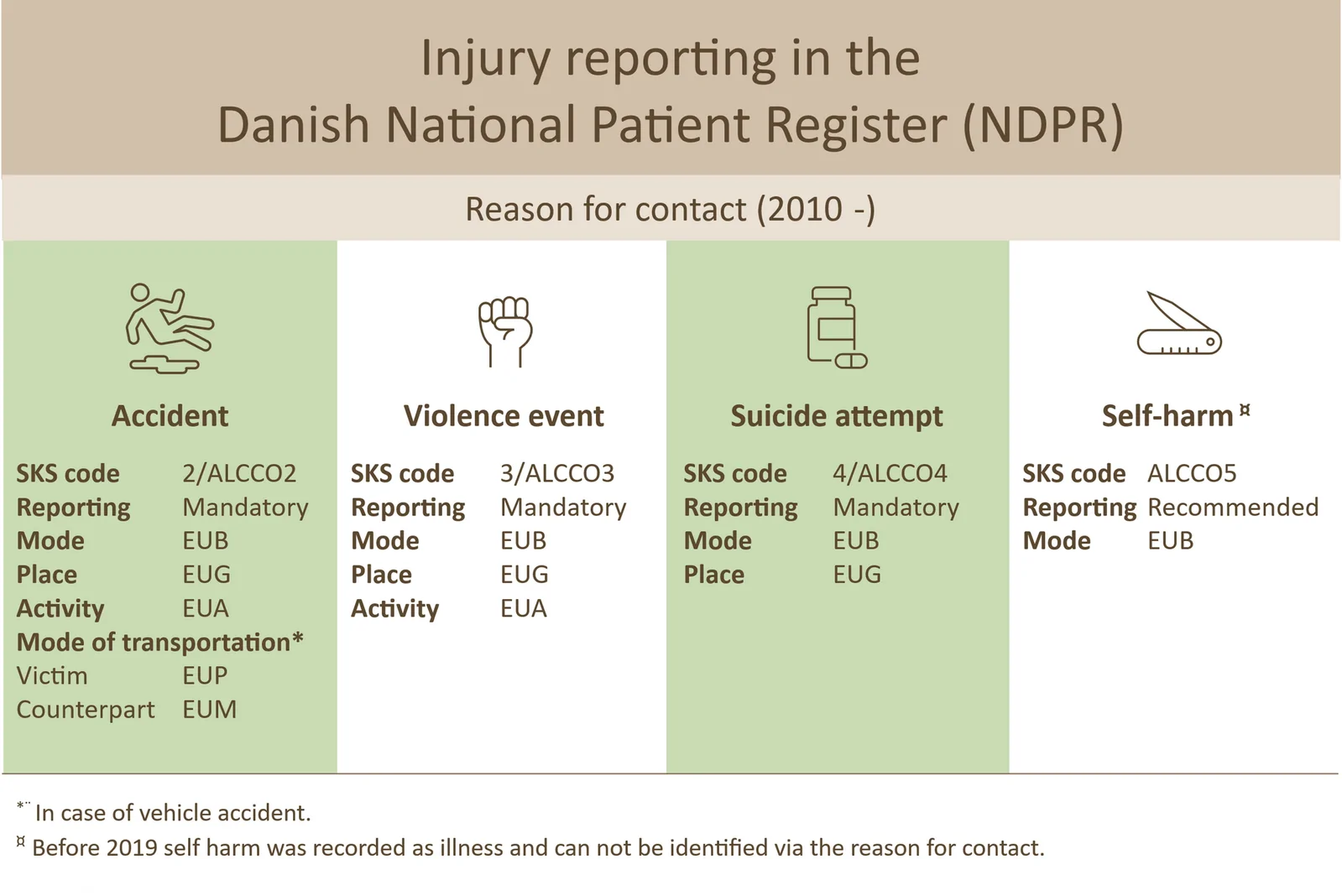
Identification of injuries reported through the DNPR. Primary contacts related to injury incidents are identified based on the recorded reason for the hospital contact. If the contact is due to an accident, an act of violence, or a suicide attempt, mandatory injury documentation is prompted using NCECI codes, specifying, for example, the mode of injury, place of occurrence, and activity involved. For transport-related accidents, the mandatory reporting also includes information on the types of transport modes involved for the injured individual and any counterpart (e.g., passenger car, pedestrian). SKS: The Danish Medical Classification System. Since 2019, voluntary injury reporting has been recommended if the contact is due to intentional self-harm.

Figure 2

shows injury reporting over time, according to the national guidelines for reporting to DNPR. Reporting the reason for contact is mandatory for all acute somatic patient contacts but must not be submitted for other types of patient encounters.

### Hospital contacts

NDIC includes all hospital contacts where the reason for contact is classified as accident, assault, or suicide/suicide attempt. From early 2019, intentional self-harm was added to the SKS codes as a distinct reason for contact and has been included in NDIC from that point onwards. Prior to 2019, intentional self-harm was recorded under illness and therefore cannot be identified via the reason for contact. Using one of the relevant reasons for contact triggers a mandatory injury report. It is possible to code any injury incident in much greater detail than illustrated in Fig. 2 using NCECI codes; however, this is not mandatory, and coding practices may vary significantly across hospitals and departments.

As a patient may appear multiple times in DNPR in relation to the same injury incident, a single key contact is identified and retained for each incident. Multiple contacts regarding the same injury incident will subsequently be linked to form individual incident trajectories. Patients may be registered with multiple injury incidents over time, which allows for further analysis of patterns across repeated events.

NDIC includes all injuries with an identified key contact that meet either of the criteria described below.

- Injury reporting (any NCECI code, mandatory or optional).
- Reason for contact listed as an accident, violence event, suicide attempt, or intentional self-harm.
- Provides an action diagnosis in the ranges DX60–DX99 or DY0–DY09.

Further details are provided in the supplementary information on operational procedures for injury incident data extraction.

### Fatalities

Fatalities for which the manner of death has been classified as accident, suicide, or violence/homicide are included.

### Explanatory variables related to injury incidents

To enable characterisation of the injury cohort all individuals registered in the NDIC are initially linked to population statistics [20] to obtain information on sex, age, and region of residence. In addition, data are linked to education registers [21] and labour market and income registers [22] to derive information on highest attained level of education and current employment status.

Sex, age and level of education are recorded at the time of the injury or death, while region of residence and employment status, is based on annual updates from population statistics referring to the individual’s status as of 31 December in the year before the injury incident or death. If not available, residence in the year of the incident was used instead, and alternatively the year after. If data on residence was not available at any of these time point, it indicates that the person was not a permanent resident of Denmark at the time of the injury incident and they were excluded from the analysis of place of residence.

### Applications and research potential of the cohort

NDIC constitutes a robust national infrastructure that supports comprehensive research within injury epidemiology and related fields. Its nationwide coverage enables analyses of temporal and regional trends in injury incidence and injury severity, as well as examinations of sociodemographic and socio‑economic disparities. Beyond descriptive surveillance, the cohort supports hypothesis‑driven studies aimed at identifying risk factors for specific injury types and elucidating underlying mechanisms. Linkage to national health and administrative registries further facilitates assessment of health, social, and economic consequences following injuries.

To examine potential differences in sociodemographic characteristics among individuals involved in injury incidents, overall and annually stratified incidence rates (IRs) can be derived from NDIC registrations in combination with appropriate reference populations. When population-level statistics are available for the stratification variables of interest, these are applied as reference denominators. In situations where such population-level data are not available, a random sample of the general population from the corresponding period is used instead.

NDIC further supports analyses of demographic, geographic, and social inequalities. Through linkage via the Civil Personal Register number, additional sociodemographic and contextual information—such as immigration status, family structure, housing type, prescribed medication, general practitioner consultations, and broader healthcare utilisation—can be incorporated into analytical models. The cohort enables research across the entire life course. Examples include studies of injuries among children and adolescents, injury incidents related to alcohol use among younger and middle‑aged populations, or falls among older adults. Moreover, NDIC supports detailed analyses of specific injury mechanisms across age groups, such as traffic accidents, poisonings, drownings, burns, sports‑related injuries, occupational injuries, and injuries associated with specific consumer products. Action diagnoses ensure accurate classification of injury type and severity, providing a solid foundation for estimating IRs, assessing geographic variation, and investigating social inequalities.

Through its longitudinal structure, NDIC supports at least one year of follow‑up after injury incidents, enabling analyses of subsequent hospital contacts, healthcare utilisation, long‑term morbidity, disability, and mortality. This follow-up period furthermore allows evaluation of preventive interventions and identification of determinants of injury risk. Advanced analytical approaches—including survival analysis for time-to-event outcomes, causal inference methods to assess the impact of interventions, and prediction modelling for identifying high-risk populations—can be applied to address complex research questions. The size and representativeness of the cohort facilitate stratified analyses across key demographic and socio‑economic groups, thereby supporting evidence‑based policy development and targeted prevention strategies.

NDIC is therefore well suited for both descriptive and analytical studies, ranging from foundational epidemiological assessments to sophisticated modelling studies, and provides a comprehensive evidence base for strengthening injury prevention and public health. While NDIC offers extensive national coverage and rich longitudinal follow‑up, certain limitations inherent to registry‑based data should be taken into account when interpreting findings.

The cohort is currently updated through 2022, and ongoing maintenance efforts aim to ensure future updates on an annual or biennial basis, thereby preserving the long‑term utility of NDIC for injury research and public health surveillance.

### Statistical analyses

All data handling was conducted in R version 4.5.2 (R Foundation for Statistical Computing) [27], using the tidyverse package collection [28].

IRs per calendar year were calculated by dividing the number of new events by the mid‑year population, an accepted approximation of total person‑time in large national cohorts [29]. Data on the overall population and stratified subgroups were obtained from population statistics provided by Statistics Denmark [30]. Exact 95% confidence intervals were derived from the Poisson distribution using chi‑square limits in accordance with standard methods [31].

IRs are presented with 95% confidence intervals; however, owing to the large underlying denominators in most analyses, these intervals are exceedingly narrow and therefore not visible at the plotted scale. Only in the mortality analyses, where event counts are lower, do the confidence intervals appear with appreciable width.

## Findings to date

During the study period from 2010 to 2022, we identified 7,217,351 primary injury contacts and 26,871 injury-related deaths for inclusion in NDIC.

In the following, we present descriptive IRs to quantify the occurrence of injuries and injury‑related mortality in the Danish population over time. Rates are shown overall and separately for men and women. For injury occurrence, IRs are further stratified by sex and age, and by region of residence to examine demographic and geographical variation.

### Population incidence of injuries and injury-related deaths

**Fig. 3:**
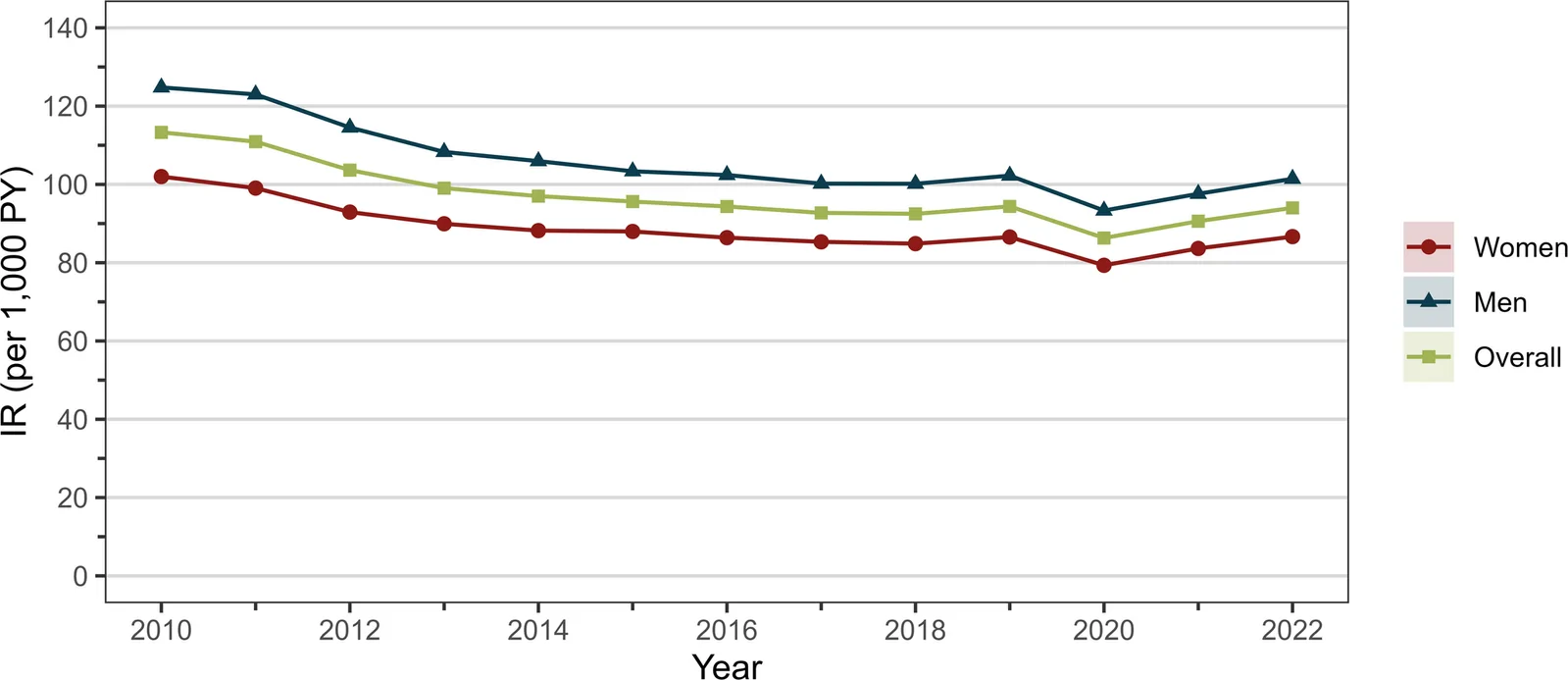
Annual incidence rates (IR) per 1,000 person-years (PY) of injury-related hospital contacts in Denmark, 2010–2022. IRs were estimated allover and separately for men and women, using mid-year population denominators for each calendar year. Point estimates are presented with corresponding 95% confidence intervals

Figure 3 depicts temporal trends in injury IRs (per 1,000 person-years) in the Danish population from 2010 to 2022. Across the entire study period the pattern of IRs were very similar for men and women. However, men consistently exhibited higher IRs than women, with men being 16.7% to 24.2% more likely to sustain an injury. Overall IRs declined gradually from 113.3 in 2010 to 94.4 in 2019, followed by a decrease in 2020 coinciding with the onset of the COVID‑19 pandemic. Rates subsequently increased in the years that followed.

**Fig. 4:**
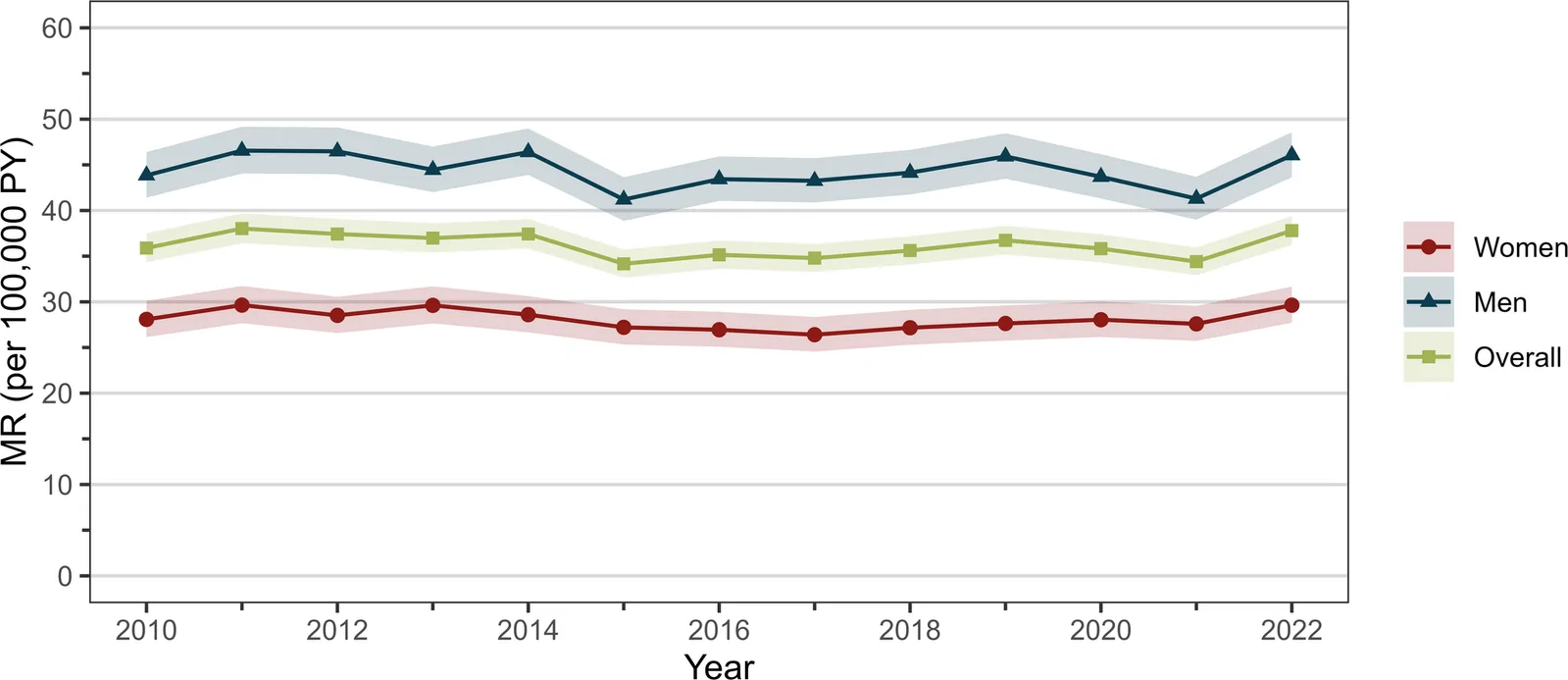
Annual mortality rates (MR) per 100,000 person-years (PY) of injury-related deaths in Denmark, 2010–2022. MRs were estimated allover and separately for men and women, using mid-year population denominators for each calendar year. Point estimates are presented with corresponding 95% confidence intervals

Injury-related mortality rates across the study period are presented in Fig. 4. Again, men consistently exhibited higher rates than women, with men being 49.7% to 66.2% more likely to die as a consequence of an injury incident. The overall mortality rates remained relatively stable throughout the study period (34.2 to 38.0 deaths per 100,000 person-years).

### IRs by sex and age

**Fig. 5:**
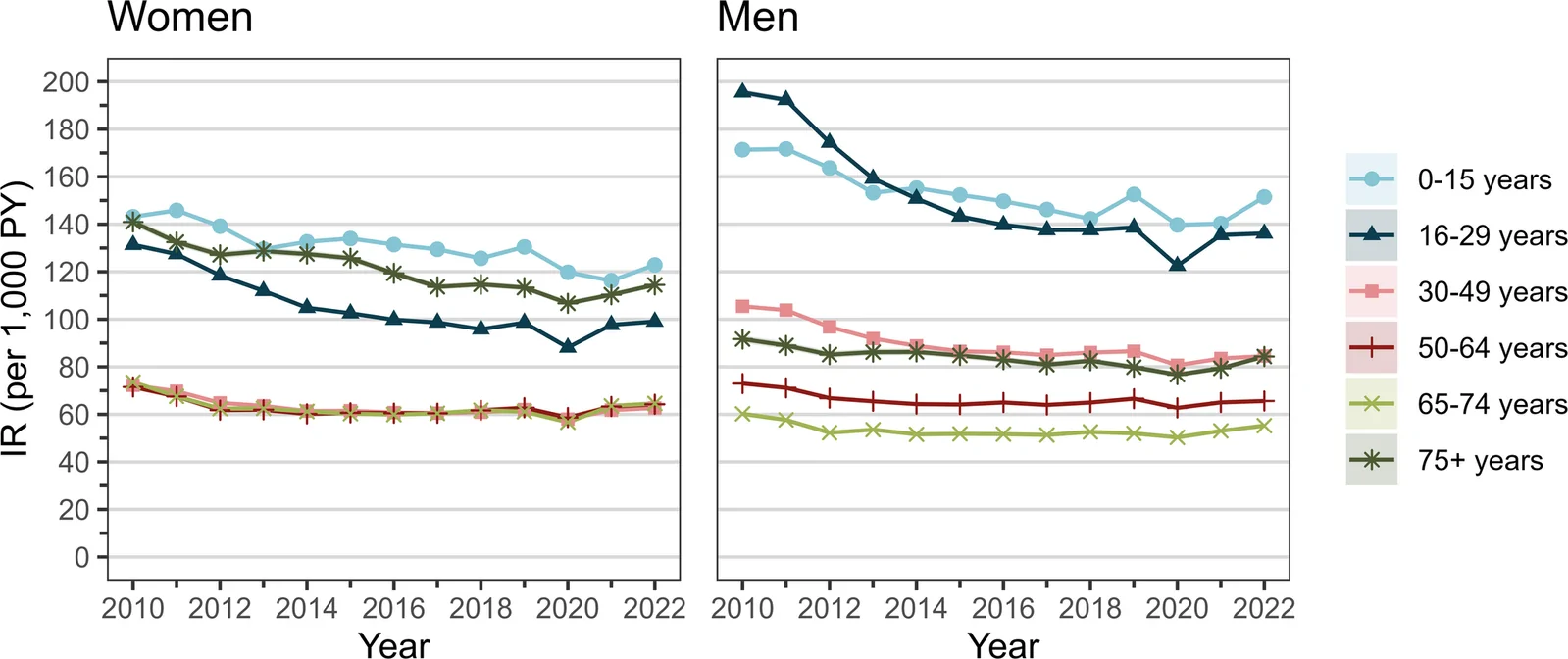
Annual incidence rates (IR) per 1,000 person-years (PY) of injury-related hospital contacts in Denmark, 2010–2022. IRs were estimated stratified by sex and age, using mid-year population denominators for each calendar year. Point estimates are presented with corresponding 95% confidence intervals

As shown in Fig. 3, men were consistently more likely to incur an injury resulting in a hospital contact than women. When further stratified by age, IRs (per 1000 person‑years) showed clear sex‑ and age‑specific differences across the study period (Fig. 5).

Among women, IRs were highest in the 0–15‑year group (116.3–145.9), followed by the ≥ 75-year group (106.7–141.0) and the 16–29-year group (88.1–131.3). All three groups exhibited declining IRs over time, and those aged 16–29 years showed a marked drop in 2020 coinciding with the COVID‑19 pandemic. Women aged 30–74 years had substantially lower and largely stable IRs, remaining between 56.7 and 64.8 from 2012 onwards after a modest decline from 2010 to 2012.

Likewise, among men, the youngest age groups had the highest IRs. Boys aged 0–15 years ranged from 139.7 to 171.7 and represented the group with the highest IR in 2022 (151.5). Men aged 16–29 years had the highest IR overall in 2010 (195.5), followed by a decline of 26.8% to 143.2 in 2015 and remaining relatively stable thereafter (aside from a marked reduction during the COVID-19 pandemic).

Adult men had lower IRs with limited temporal variation in the 30–49‑year group (80.7–105.5), and in the 50–64‑year group (62.7–73.0). IRs were lowest and most stable among older men between 65 and 74 years of age (50.3–60.2), and for those aged ≥ 75 years (76.7–91.7).

Overall, men had higher IRs across all age groups except those aged ≥ 65 years. IRs were similar between sexes for individuals aged 65–74 years, whereas women aged ≥ 75 years consistently exhibited higher IRs than men.

### IRs by place of residence

**Fig. 6:**
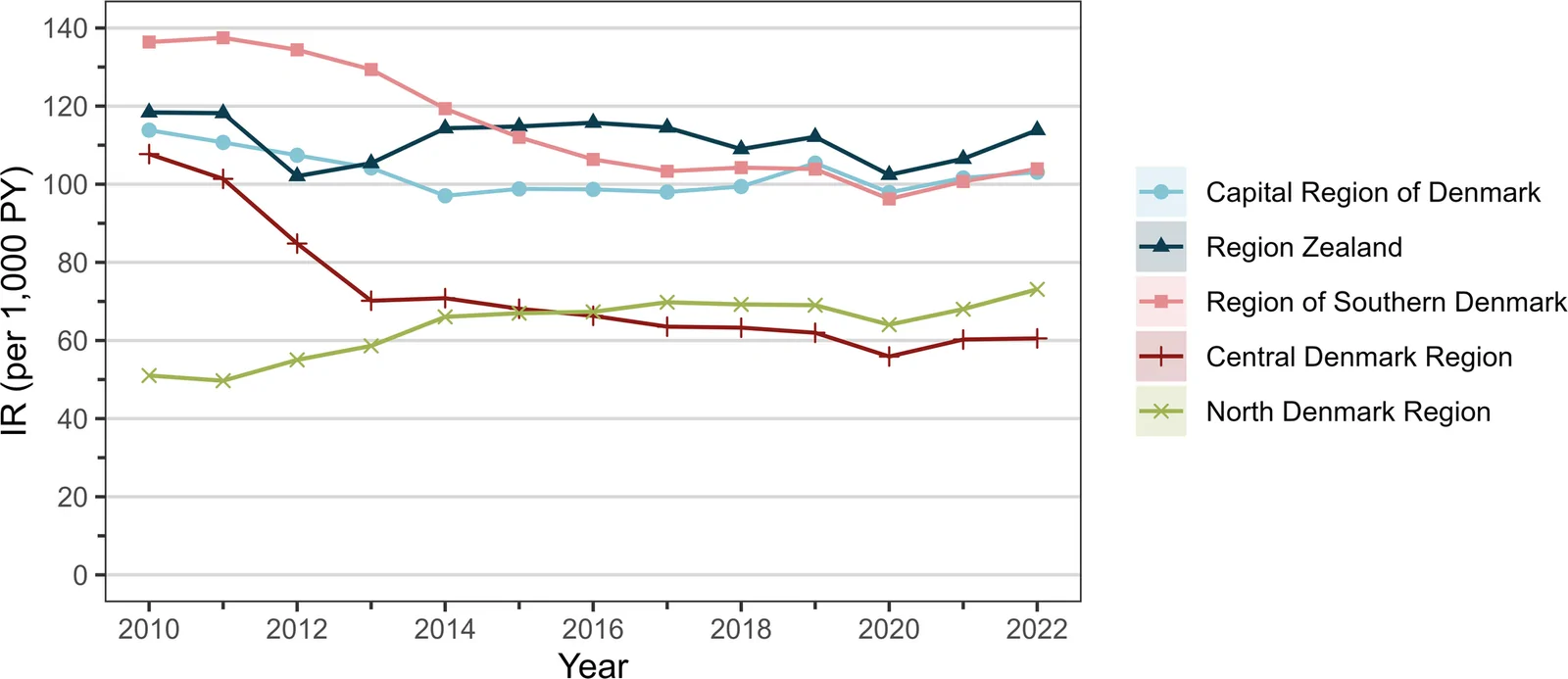
Annual incidence rates (IR) per 1,000 person-years (PY) of injury-related hospital contacts in Denmark, 2010–2022. IRs were estimated allover and separately for the populations in each of the five Danish regions, using mid-year population denominators for each calendar year. Point estimates are presented with corresponding 95% confidence intervals

With respect to region of residence, Fig. 6 shows that IRs of injuries resulting in a hospital contact (per 1,000 person‑years) declined between 2010 and 2014 in the Region of Southern Denmark (from 136.4 to 119.4), the Capital Region of Denmark (from 113.8 to 97.0), and the Central Denmark Region (from 107.7 to 70.8). In 2010, the IRs were 136.4, 113.8, and 107.7, respectively; by 2014, the IR in the Region of Southern Denmark had decreased to 112.0.

In contrast, the North Denmark Region experienced an increase during the same periode (from 51.0 to 66.0). In Region Zealand, IRs fell from 2010 to 2012 (from 118.4 to 102.1) but then rose again to approximately the 2010 level by 2014.

From 2015 onwards, IRs in all regions were relatively stable with minor fluctuations. In 2022, the lowest IRs occurred in the Central Denmark Region and the North Denmark Region (60.5–73.1), whereas the highest were in the Region Zealand (113.8) and in the Region of Southern Denmark and the Capital Region of Denmark (103.9–103.1).

Across the study period, the lowest IRs were consistently observed in the North Denmark Region and the Central Denmark Region.

## Discussion of the data foundation

### Strengths of the data

A strength of this study is the use of national, population‑based register data, providing nationwide coverage of hospital‑registered injury incidents and injury‑related deaths within the Danish healthcare system. Because NDIC integrates information across all hospital contacts nationwide, the database is largely free from the selection biases typically encountered in studies based on restricted cohorts or survey samples. The standardised diagnostic coding systems (ICD‑10 and NCECI) support consistent and accurate classification of injury incidents across calendar years, thereby strengthening internal validity and facilitating stable temporal analyses. The large sample size provides substantial statistical power, enabling robust estimation of IRs, examination of complex interactions, and testing of nuanced hypotheses. The national coverage also enhances external validity, ensuring that findings are generalisable to the Danish population, although they remain contingent on patterns of healthcare utilisation and does not capture injuries not resulting in hospital contact or death.

### Limitations of the data

However, several important limitations must be acknowledged. NDIC only captures injury incidents that result in a hospital contact, meaning that injuries not leading to hospital attendance—particularly minor events—are not included. Consequently, care‑seeking behaviour may introduce residual selection into the dataset, and such patterns may vary geographically, for example in relation to distance to hospitals or emergency departments [8]. In registry‑based research, there is an inherent risk of misclassification, including variation in diagnostic and coding practices across clinicians, hospitals, and time periods, which may affect the quality, consistency, and completeness of registered injury incidents. Register data also provide limited information on behavioural, contextual, and situational factors, constraining adjustment for confounders and limiting detailed mechanistic interpretation. Finally, updates to underlying registers may entail temporal delays. Taken together, these limitations underscore the need for continuous methodological refinement and critical reflection on the data sources used, and they reflect the classic register‑based trade‑off: strong population coverage, completeness, and robust longitudinal follow‑up, but limited detail on behaviour, context, and exposure mechanisms.

### Organisation of the healthcare system

Not all individuals receive treatment within the hospital system after an injury incident, which is a prerequisite for inclusion in NDIC. A previous study based on self-reported injuries showed that only about half of all injuries were treated in a hospital setting [11]. Some individuals are instead managed by general practitioners, through public or private out-of-hours medical services, or receive on-site prehospital treatment without hospital transport, while others cope without any professional assistance. Where an injured person seeks care—or is referred to—depends on the organisation of healthcare services in the given area. In regions with long distances to emergency departments or acute care clinics, fewer injured individuals will use these services compared to areas with easier access, even if the incidence of injuries is the same [32]. Hospital admission rates are less affected by such differences, as admissions typically involve more severe injuries requiring hospital-based treatment.

### Coding practices

When a patient is admitted to hospital following an injury incident, healthcare staff routinely inquire about the circumstances of the injury incident. The primary questions cover; reason for contact (whether the injury was caused by an accident, an act of violence, a suicide attempt, or other intentional harm), mode of injury (fall, poisoning, burn, blow, impact, etc.), location of injury (home, road, institution such as daycare or school, etc.), and activity at the time of injury (paid work, sports, etc.).

For traffic‑related incidents, staff additionally record the patient’s and any counterpart’s mode of transport (e.g. bicycle, pedestrian, passenger car). Although this framework is standardised, there is considerable variation in how thoroughly these questions are recorded, which directly affects the completeness and detail of the data.

The extent and quality of coding depend on local workflows, staffing resources, training practices, and the priority placed on complete registration. While some hospitals employ structured routines for documenting external causes of injury—ensuring that patients receive detailed EU‑codes alongside relevant contextual variables—others routinely record only the mandatory elements. Furthermore, the accuracy of the recorded information may vary, as the level of detail and precision applied during coding differs across institutions.

Importantly, coding is constrained by the available categories within the coding systems, meaning that coders have limited options for capturing relevant circumstances, not necessarily because the information was unavailable, but because the coding framework lacks adequate categories [submitted paper, Kruscow 2026]. Such structural limitations can generate systematic differences in the level of detail of contextual data across regions and institutions, even when the underlying injury burden is comparable.

Miscoding may arise from ambiguous clinical presentations, unclear injury narratives, or inconsistencies in how clinicians interpret coding guidelines. Missing codes may result from incomplete documentation or from clinical prioritisation. Both miscoding and missing coding can contribute to information bias, particularly when comparing subgroups that differ in care‑seeking patterns, hospital workflows, or local resource availability.

### Use of standardised codes

The application of standardised coding systems, including ICD‑10 [25] for diagnoses and NCECI codes for external causes [25], provides a coherent and uniform framework for documenting injury incidents This standardisation enhances consistency across years, settings, and regions by reducing variation arising from local terminology or heterogeneous documentation practices. Furthermore, the use of internationally recognised coding systems facilitates meaningful comparison with injury surveillance data from other countries, thereby supporting cross‑national analyses and strengthening the broader evidence base within injury epidemiology. Although these systems provide a robust structural framework, the fidelity with which they are applied in routine clinical practice remains a key source of variation.

## Perspectives/future plans

Injury prevention is a critical public health priority that intersects with major agendas such as socioeconomic health inequities, economics, the mental health crisis, and climate change, and spans multiple sectors, including healthcare, resilience and preparedness, traffic, labour markets, and the criminal justice system. For example, self-harm and suicide are closely linked to the mental health crisis, while unintentional injuries caused by extreme weather events are projected to increase in the coming decades. Violent acts not only lead to severe health consequences but also impose considerable economic and administrative demands on the justice system.

This makes injury prevention not only a health imperative but also a strategic lever for achieving broader societal gains. Effective prevention yields benefit across multiple sectors, reduces social inequalities, and strengthens outcomes for individuals, their relatives and society at large. Moreover, systematic surveillance provides a critical foundation for evaluating the effectiveness of prevention efforts and guiding continuous improvement.

This cohort offers substantial potential to contribute to key research areas across the lifespan and to identify targeted prevention opportunities at each stage of the life course. During childhood and adolescence, unintentional injuries remain a leading cause of morbidity and mortality, highlighting the need for early safety and behavioural interventions. Among working-age adults, workplace accidents constitute a major concern with implications for long-term health, economic stability, and productivity. In older adults, falls represent one of the most significant contributors to functional decline and healthcare utilisation, and downstream care costs, pointing to important opportunities for environmental, medical, and rehabilitative prevention strategies. By enabling comparative analyses across age groups, this cohort allows for the identification of age-specific risk patterns and the evaluation of interventions tailored to distinct life stages.

To reduce preventable fatalities, the substantial burden of serious non‑fatal injuries—including accidents, acts of violence, suicide attempts, and self‑harm—that result in temporary incapacity and long‑term disability considerable societal and economic costs underscores the need for continuous and systematic surveillance of injury trends. NDIC is uniquely positioned to support such efforts and to inform future prevention strategies that are evidence-based, equity‑oriented, and responsive to changes across the population.

## Supplementary Information

Below is the link to the electronic supplementary material.

Supplementary Material 1

## Abbreviations

NDIC: The national Danish injury cohort
DNPR: The Danish national patient register
ICD: The international classification of diseases
NOMESCO: The nordic medico-statistical committee
NCECI: The NOMESCO classification of external causes of injuries
SKS: The Danish medical classification system
IDB-EU: The European injury data base
IR: Incidence rate
