# Supplementary material for: Design and characteristics of the national Danish injury cohort (NDIC)
Source: Eur J Epidemiol. 2026 Jun 8;41(7):907–18. doi: 10.1007/s10654-026-01413-2 (PMC13572329; doi:10.1007/s10654-026-01413-2)
Supplement: Supplementary file 1 [file 10654_2026_1413_MOESM1_ESM.docx]

# SUPPLEMENTARY INFORMATION

## Operational procedures for injury incident data extraction

### Registration framework for injury incident data in DNPR

During the study period from 2010 ongoing, two versions of the Danish National Patient Register (DNPR) has been used [23].

In early 2019, version 2 of the Danish National Patient Register (DNPR‑2) was replaced by version 3 (DNPR‑3), which introduced changes to both reporting structures and data extraction procedures.

In DNPR‑2, an accident table was generated within the register based on reported codes. This table serves as a key component in the identification of accidents in Denmark, as it contains contextual information on injury incidents leading to hospital contact in the form of EU‑codes. In DNPR‑3, this table is replaced by a results table. Injury incidents are reported using a dedicated result reporting type, which enables the isolation of injury‑related records directly through this code. The NDIC includes all hospital contacts recorded in the former accident table as well as all injury results reported through the results table.

While the accident table is not available for private hospitals—due to the absence of private emergency departments or urgent care clinics—the results table is available across sectors.

The table below summarises the differences in the availability of injury incident data between DNPR‑2 and DNPR‑3.

**Supplementary table 1** Available tables and variables relevant to injury incidents in DNRP-2 and DNPR-3.

| **Information** | **DNPR-2** |  | **DNPR-3** |  |
| --- | --- | --- | --- | --- |
|  | **Table** | **Variable** | **Table** | **Variable** |
| Reason for contact | LPR_ADM | C_KONTAARS | LPR_A_KONTAKT | KONT_AARSAG |
| Acute status | LPR_ADM | C_INDM | LPR_A_KONTAKT | PRIORITET |
| Type of contact | LPR_ADM | C_PATTYPE | LPR_A_KONTAKT | KONT_TYPE |
| Action diagnosis | LPR_ADM | C_ADIAG | LPR_A_KONTAKT | ADIAG |
| Accident reporting | LPR_ULYK |  | LPR_A_RESULTATER | RES_TYPE |
| NCECI codes | LPR_ULYK | C_ULYK | LPR_A_RESULTATER | RES_VAERDI_TEKST |

### Reproducible identification of injury-related hospital contacts

NDIC contains information on hospital treated juries in Denmark, regardless of permanent residency. In contrast injury-related deaths are only available for people with permanent residency in Denmark.

First, all potentially injury-related hospital contacts were identified. A contact was included if it fulfilled any one of the following criteria:

- Reason for contact: 2, 3, or 4 (in DNPR-2), or ALCCCO2, ALCCCO3, ALCCCO4, or ALCCCO5 (in DNPR-3).
- Action diagnosis: DS00-DT99, or DX60-DY09.
- Injury reporting: In the accident table (in DNPR-2), or a result reporting type RAA05 in the result table (in DNPR-3).

To identify key contacts the criteria below was applied to all injury-related hospital contacts. A key contact was identified if it fulfilled any one of the following criteria:

- Injury reporting: contacts with at least one, mandatory or optional, SKS EU-code (NCECI code).
- Reason for contact listed as an accident (2 or ALCCCO2), violence event (3 or ALCCCO3), suicide attempt (4 or ALCCCO4), or intentional self-harm (ALCCCO5).
- Action diagnosis in the ranges
  - DX60-DX84 (intentional self-harm)
  - DX85–DX99 or DY0–DY09 (violence events).

**References**

23. Sundhedsdatastyrelsen. Landspatientregisteret [The danish national patient register]. <https://sundhedsdatastyrelsen.dk/data-og-registre/nationale-sundhedsregistre/landspatientregisteret>. Accessed 02/20/2026.
